# Investigating the association between hyperthyroidism and the risk of herpes zoster in a cohort study in Taiwan

**DOI:** 10.37796/2211-8039.1664

**Published:** 2025-09-01

**Authors:** Shih-Wei Lai, Yu-Hung Kuo, Kuan-Fu Liao

**Affiliations:** a School of Medicine, China Medical University, Taichung, Taiwan; b Department of Family Medicine, China Medical University Hospital, Taichung, Taiwan; c Department of Research, Taichung Tzu Chi Hospital, Taichung, Taiwan; d College of Medicine, Tzu Chi University, Hualien, Taiwan; e Division of Hepatogastroenterology, Department of Internal Medicine, Taichung Tzu Chi Hospital, Taichung, Taiwan

**Keywords:** Cohort, Herpes zoster, Hyperthyroidism, Taiwan

## Abstract

**Background:**

The aim of this cohort study was to investigate the association between hyperthyroidism and the likelihood of developing herpes zoster in Taiwan.

**Methods:**

Using the National Health Insurance Research Database (NHIRD) in Taiwan, we selected individuals aged 20–84 who were newly diagnosed with hyperthyroidism between 2013 and 2020 as the hyperthyroidism group. These individuals were then matched with a control group without hyperthyroidism in a 1:1 propensity score matching for sex, age, and baseline comorbidities. The occurrence of herpes zoster was tracked in both groups until the end of the study period or until a diagnosis of herpes zoster was made. The Cox proportional hazards regression analysis was used to determine the hazard ratio (HR) and 95 % confidence interval (CI) for the risk of herpes zoster associated with hyperthyroidism.

**Results:**

A total of 202,069 individuals with hyperthyroidism and 202,069 individuals without hyperthyroidism were included in the analysis. The incidence rate of herpes zoster was higher in the hyperthyroidism group compared to the non-hyperthyroidism group (6.10 per 1000 person-years for the hyperthyroidism group versus 5.53 per 1000 person-years for the non-hyperthyroidism group, incidence rate ratio = 1.10, 95 %CI = 1.07–1.14, and P value < 0.001). After adjusting for covariables, individuals with hyperthyroidism were found to have a higher risk of developing herpes zoster compared to those in the non-hyperthyroidism group (adjusted HR = 1.19, 95 %CI = 1.15–1.23, and *P* < 0.001).

**Conclusion:**

This cohort study suggests that individuals with hyperthyroidism in Taiwan may have a greater risk of developing herpes zoster compared to those without hyperthyroidism.

## 1. Introduction

Herpes zoster occurs due to the reactivation of the varicella-zoster virus, which remains dormant in dorsal root ganglia after primary infection (chickenpox) [1–4]. Herpes zoster typically presents with painful, unilateral grouped vesicles on an erythematous base and it can lead to serious complications such as postherpetic neuralgia, especially in individuals ≥50 years old and in immunocompromised individuals [1–4]. Understanding the risk factors for herpes zoster is critical for developing effective prevention and management strategies. In addition to older age, numerous chronic conditions, such as cardiovascular diseases, chronic kidney disease, inflammatory bowel disease, chronic pancreatitis, psychological stress, and others, have been found to be associated with an increased risk of herpes zoster [5–10]. However, hyperthyroidism has not yet been studied in this field.

Previous research has explored the association between endocrine and autoimmune disorders and the risk of herpes zoster [11–16]. Hyperthyroidism is a state in which there is an overproduction of thyroid hormones. Autoimmune disorders like Graves’ disease involve considerable changes in the immune system [17–22]. Autoimmune responses can lead to different kinds of long-term inflammation and disturbances in the immune system [23–25], which can impede the body’s defense against infections. The immune system, especially cell-mediated immunity, is crucial in keeping the varicella-zoster virus inactive [26–28]. Impairments in the immune system can cause the virus to become active again, as seen in individuals with herpes zoster.

What is known:

1. Hyperthyroidism significantly impacts multiple physiological systems and has been linked to various forms of immune disturbances, which can impede the body’s defense against infections.
2. Numerous risk factors for herpes zoster are identified, but hyperthyroidism has not yet been studied in this field.

What is new here:

1. The incidence rate of herpes zoster was higher in individuals with hyperthyroidism compared to those in the non-hyperthyroidism group.
2. After adjusting for covariables, individuals with hyperthyroidism were found to have a higher risk of developing herpes zoster compared to those in the non-hyperthyroidism group (adjusted HR = 1.19 and 95 %CI = 1.15–1.23).

Translational impact:

1. Clinicians should be aware of the increased probability of herpes zoster in individuals with hyperthyroidism.
2. Preventive strategies, such as the administration of the herpes zoster vaccine, might be beneficial in individuals with hyperthyroidism.

Considering the influence of thyroid hormones on immune regulation, it’s reasonable to suggest a connection between hyperthyroidism and an increased occurrence of herpes zoster. Current research focusing on the correlation between hyperthyroidism and the likelihood of herpes zoster occurrences is little. The purpose of this cohort study is to explore this potential correlation by analyzing data from individuals diagnosed with hyperthyroidism and comparing their incidence of herpes zoster with a control group without hyperthyroidism. The findings can potentially contribute to enhancing clinical protocols and guidelines for managing individuals with hyperthyroidism, potentially leading to improved preventive strategies.

## 2. Methods

### 2.1. Study design and data source

**Fig. 1:**
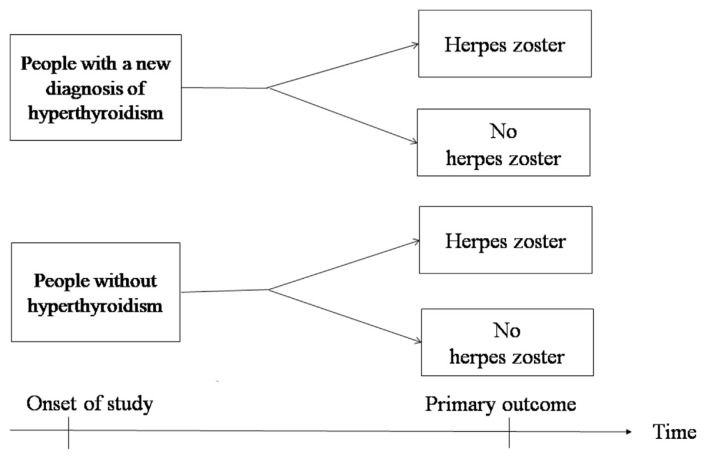
The concept of this cohort study.

The null hypothesis is that there is no significant correlation between hyperthyroidism and the development of herpes zoster. The alternative hypothesis is that there is a significant link between hyperthyroidism and the development of herpes zoster. A retrospective cohort study was designed to examine whether the observed data support the null hypothesis or the alternative hypothesis in the study population (Fig. 1).

The data source was the claims data from the National Health Insurance Research Database (NHIRD) in Taiwan, spanning the period from 2013 to 2020. This dataset comprises medical records of outpatient visits, hospitalizations, emergency department visits, and medication utilization.

### 2.2. Study subjects

**Fig. 2:**
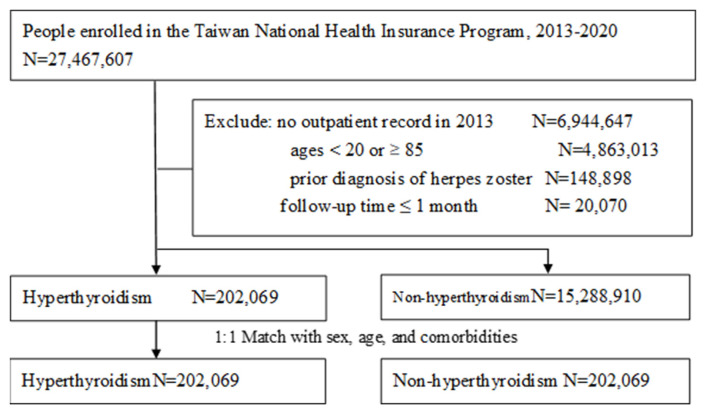
Algorithmic approach to selection of study subjects.

The hyperthyroidism group consisted of individuals aged 20–84 who were newly diagnosed with hyperthyroidism, identified by the International Classification of Diseases codes: ICD-9 codes 242 and ICD-10 codes E05. The index date was defined as the date of hyperthyroidism being diagnosed. For each individual in the hyperthyroidism group, one individual without a diagnosis of hyperthyroidism was randomly selected to form the non-hyperthyroidism group. Both groups were matched by sex, age, and the baseline comorbidities using 1:1 propensity score matching (Fig. 2). The diagnoses for all baseline comorbidities were made by utilizing both ICD-9 and ICD-10 coding systems. The index date for the non-hyperthyroidism group was defined as the date of their first outpatient visit in 2013. To confirm that there were medical records in 2013, individuals who did not have any outpatient visits in 2013 were excluded from the study. Individuals previously diagnosed with herpes zoster before the index date were not included in the study. Also, the study did not incorporate individuals who had a follow-up duration less than one month. The data related to previous immunization against herpes zoster was not included as a variable in this study.

### 2.3. Primary outcome

The primary outcome of the cohort study was the detection of new cases of herpes zoster during the observation period, identified by ICD-9 codes 053 and ICD-10 codes B02. All individuals were monitored until they were diagnosed with herpes zoster or until the end of the research duration in 2020.

### 2.4. Statistical analysis

In order to analyze differences in categorical variables between the hyperthyroidism and non-hyperthyroidism groups, a Chi-square test was applied. For examining dissimilarities in continuous variables between the two groups, we employed a t-test. The cumulative incidence of herpes zoster in both the group with hyperthyroidism and the group without was depicted using a Kaplan-Meier curve over the duration of the follow-up. To compare the differences in herpes zoster occurrence between the two groups, a log-rank test was carried out. The incidence density of herpes zoster was calculated by dividing the count of herpes zoster occurrences identified throughout the observation period by the total person-years of follow-up for each group. We validated the assumption of proportionate risks using a test of scaled Schoenfeld residuals, which confirmed its validity. After accounting for various contributing factors, we employed a multivariable Cox proportional hazards regression analysis to examine the correlation between various factors and the likelihood of herpes zoster.

The measure of the association’s intensity was presented using both the hazard ratio (HR) and the respective 95 % confidence interval (CI). We established a P-value of 0.05 as the benchmark for determining statistical significance, and considered any value less than this as indicative of a significant statistical discrepancy. The SAS software was used in all analyses (version 9.4 for Windows; SAS Institute Inc., Cary, NC, USA).

## 3. Results

### 3.1. Characteristics of study subjects

The characteristics of the study subjects are shown in Table 1. The cohort study included 202,069 individuals in the hyperthyroidism group and 202,069 individuals in the non-hyperthyroidism group. Study subjects had a mean age of 46 years and 24.7 % were male subjects in both the hyperthyroidism and the non-hyperthyroidism groups. Based on well-matching on the baseline comorbidities through 1:1 propensity score matching, there were no differences in the proportions of the baseline comorbidities between the two groups.

The mean observation period was 4.1 years for the hyperthyroidism group and 7.5 years for those without hyperthyroidism. The disparity in the average length of follow-up implies that those without hyperthyroidism were tracked for a more extended period, on average, than those in the hyperthyroidism group (as per the t-test, *P* value < 0.001).

### 3.2. Incidence density of herpes zoster

In Table 2, the incidence rate of herpes zoster was higher in the hyperthyroidism group compared to the non-hyperthyroidism group (6.10 per 1000 person-years for hyperthyroidism group versus 5.53 per 1000 person-years for non-hyperthyroidism group, incidence rate ratio = 1.10, 95 %CI = 1.07–1.14, and *P* value < 0.001). When stratified by sex and age groups, the incidence rates of herpes zoster were consistently higher in the hyperthyroidism group compared to the non-hyperthyroidism group.

Individuals aged 65–84 in the hyperthyroidism group had the highest rate of herpes zoster (14.42 per 1000 person-years).

**Fig. 3:**
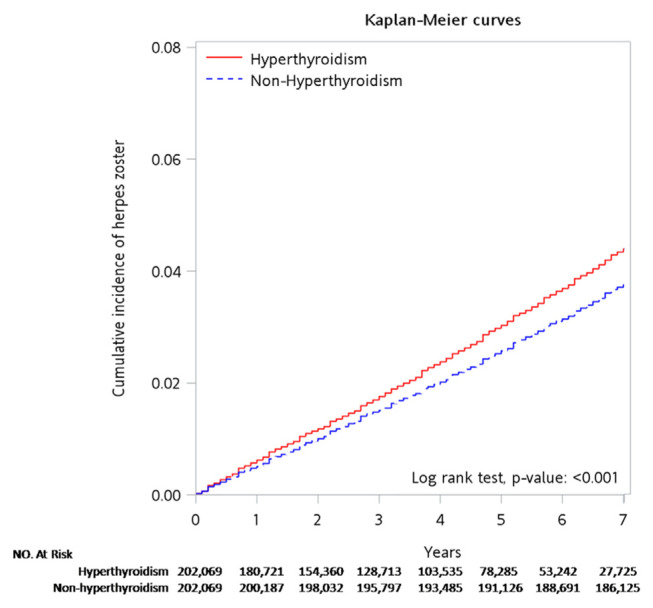
Kaplan-Meier model demonstrates the cumulative incidence of herpes zoster for the hyperthyroidism group and the non-hyperthyroidism group over the follow-up period (P < 0.001).

In Fig. 3, according to the Kaplan-Meier curve, the hyperthyroidism group experienced a higher cumulative incidence of herpes zoster than the non-hyperthyroidism group across the entire observation period (*P* value < 0.001). This finding indicates a notable statistical difference in the occurrence of herpes zoster between the two groups.

### 3.3. Association between hyperthyroidism and the likelihood of herpes zoster

Initially variables showing a significant association with herpes zoster in an unadjusted model were selected for consideration in an adjusted model. This was aimed at assessing their independent influences after controlling for confounding variables (Table 3). After controlling for confounding variables including sex, age, cerebrovascular disease, chronic kidney disease, chronic obstructive pulmonary disease, coronary artery disease, diabetes mellitus, hyperlipidemia, and hypertension, the multivariable Cox proportional hazards regression model revealed a HR of 1.19 (with a 95 %CI of 1.15–1.23 and *P* < 0.001) for the risk of herpes zoster among the hyperthyroidism group compared to the non-hyperthyroidism group. These results suggest a positive association between hyperthyroidism and an increased chance of acquiring herpes zoster, pointing out that individuals with hyperthyroidism bear a higher risk for herpes zoster. Moreover, the 95 % confidence interval offers a plausible range for the true hazard ratio, demonstrating that it could potentially be as small as 1.15 or as elevated as 1.23, with a 95% level of confidence. The limited range of the confidence interval adds strength to the conclusion of a positive correlation between hyperthyroidism and an increased susceptibility to herpes zoster.

## 4. Discussion

This cohort study revealed a notable link between hyperthyroidism and a higher chance of contracting herpes zoster. Notably, the hyperthyroidism group displayed a higher incidence rate of herpes zoster than the non-hyperthyroidism group, indicated by incidence rates of 6.10 and 5.53 per 1000 person-years respectively. This heightened risk was maintained even after controlling for different variables, demonstrating an adjusted hazard ratio of 1.19 (95 % CI = 1.15–1.23). Consequently, these findings propose that individuals with hyperthyroidism have a 19% increased likelihood of developing herpes zoster compared to those without hyperthyroidism. Previous research has explored the association between endocrine and autoimmune disorders and the risk of herpes zoster [11–16], but the link between hyperthyroidism and the risk of herpes zoster has not been explored in any other study. A direct comparison is not possible. Therefore, the findings of our cohort study provide valuable concepts into this association.

While this study does not explore the precise mechanisms responsible for the observed connection between hyperthyroidism and an increased likelihood of herpes zoster, in general, the factors causing disturbances in the immune system in hyperthyroidism are intricate and consist of both direct impacts of thyroid hormones on immune cells and indirect effects via changes in cytokine production and autoimmune processes [17–25].

It is necessary to address some clinical implications and potential limitations of the study. First, the absolute risk increase is 0.57 per 1000 person-years. Therefore, the hyperthyroidism group could potentially increase the incidence of herpes zoster by approximately 6 cases per 10,000 person-years. Therefore, in this case, the Number Needed to Harm (NNH) is approximately 1754. This means that for every 1754 individuals with hyperthyroidism over a one-year period, one additional case of herpes zoster is expected compared to individuals without hyperthyroidism. Whether the NNH of approximately 1754 is considered high or low depends on several factors and can vary based on the context and perspectives of healthcare providers, patients, and public health officials. However, this does not negate the potential benefits of preventive measures like vaccination. Vaccination can still be considered beneficial in reducing the overall burden of herpes zoster in at-risk populations, including those with hyperthyroidism, despite the high cost of herpes zoster vaccine and the specific NNH value. Clinicians should be aware of the increased probability of herpes zoster in individuals with hyperthyroidism. Preventive strategies, such as the administration of the herpes zoster vaccine, might be beneficial in individuals with hyperthyroidism. Second, the observation period for the non-hyperthyroidism group was initiated in 2013. If herpes zoster didn’t occur, the observation spanned nearly 8 years (until 2020), yielding a follow-up duration averaging 7.5 years. On the other hand, the hyperthyroidism group’s enlistment spanned from 2013 to 2020, resulting in a mean follow-up duration of 4.1 years. The follow-up period might be too short to capture all herpes zoster cases in the hyperthyroidism group, leading to right censoring. That is, this could lead to more cases of right-censored data, where individuals do not develop herpes zoster by the end of the study. In such cases, their full risk period—up until they might develop herpes zoster—is not captured within the study timeframe. As a result, the study may underestimate the actual risk of herpes zoster in the hyperthyroidism group. However, regardless of this possible underestimation, the study revealed an elevated hazard ratio for the hyperthyroidism group, signaling a significant correlation between hyperthyroidism and a heightened risk of herpes zoster development. Third, due to the inherent limitations of observational studies, our cohort research cannot confirm causality with absolute certainty. However, we propose that individuals with hyperthyroidism may be more susceptible to herpes zoster. Fourth, while adjustments were made for some potential confounding factors, the influence of variables not measured in this study, such as medication use or unmeasured factors affecting immune function and subsequent viral reactivation, cannot be completely eliminated. Fifth, using ICD-9 and ICD-10 codes as a method for diagnosing hyperthyroidism, diagnosing comorbidities, and diagnosing herpes zoster carries some limitations, including possible errors and a lack of validation through chart review. These codes might be incorrectly recorded, and the documented dates for diagnosis might not align with the actual timing when these conditions were detected. Sixth, in Taiwan, the National Health Insurance Program does not comprehensively cover the herpes zoster vaccine. Individuals who opt for the herpes zoster vaccine are responsible for paying the cost of the vaccine as well as any related administration charges. Consequently, these vaccinations might not be systematically logged in the Taiwan insurance claim systems. This study did not include prior vaccination for herpes zoster as a variable in the analysis, which can potentially present difficulties in differentiating between individuals who have been vaccinated and those who have not. Seventh, regardless of whether individuals received their herpes zoster diagnosis at another hospital, the Taiwan National Health Insurance Program’s claims data still records this diagnosis. Therefore, the chance of a follow-up loss due to overlooked diagnoses can be effectively reduced. Eighth, based on the high quality of Taiwan’s medical care system, individuals with hyperthyroidism generally have access to timely and effective treatment. This ensures that most individuals with hyperthyroidism receive appropriate management, including medications and regular monitoring, which can help mitigate complications. One key point is the absence of blood data on thyroid function tests in this study. This limitation prevents us from evaluating the thyroid status at the time of herpes zoster development. Consequently, we cannot ascertain whether individuals were still experiencing hyperthyroidism or had returned to euthyroid status when herpes zoster occurred. Understanding the thyroid status during herpes zoster development is crucial for elucidating the association between hyperthyroidism and herpes zoster. Without data on thyroid function tests, the study lacks the depth needed to fully explore the underlying mechanisms of the observed associations. Despite the limitations mentioned above, our study benefits from a substantial sample size, providing sufficient statistical power to detect significant correlations or differences, thereby enhancing the reliability of our findings. Additionally, the use of a comprehensive and longitudinal dataset from the National Health Insurance Research Database (NHIRD) in Taiwan allows for extensive follow-up and the capture of detailed medical histories. The matched cohort design helps to minimize selection bias and ensures comparability between the hyperthyroidism and non-hyperthyroidism groups.

## 5. Conclusions

Based on the findings of this cohort study, individuals with hyperthyroidism may have an approximately 19% higher likelihood of developing herpes zoster compared to those without hyperthyroidism, after controlling for confounding variables. However, it’s essential to acknowledge the limitations of this study, such as the lack of thyroid function test data, which could provide deeper insights into the mechanisms underlying this association. While further research is warranted to elucidate the underlying biological mechanisms, our findings support the consideration of preventive strategies, including vaccination, for individuals with hyperthyroidism to mitigate the risk of herpes zoster and its associated complications.

## Data availability statement

The insurance reimbursement claims data used in the study are available for public access.

## Figures and Tables

**Table 1:** Baseline information between hyperthyroidism group and non-hyperthyroidism group.

Variable	Hyperthyroidism N = 202,069		Non-hyperthyroidism N = 202,069		*P* value^a^
	
	n	(%)	n	(%)	
Sex					1.000
Male	49833	24.7	49833	24.7	
Female	152236	75.3	152236	75.3	
Age group (years)					0.988
20–39	71808	35.5	71792	35.5	
40–64	105007	52.0	105014	52.0	
65–84	25254	12.5	25263	12.5	
Age (years), mean ± standard deviation^b^	46.5 ± 14.6		46.6 ± 14.6		0.947
Follow-up time (years), mean ± standard deviation^b^	4.1 ± 2.3		7.5 ± 1.3		<0.001
Baseline comorbidities					
Alcohol-related disease	665	0.3	649	0.3	0.658
Cerebrovascular disease	6346	3.1	6345	3.1	0.993
Chronic kidney disease	4217	2.1	4211	2.1	0.947
Chronic obstructive pulmonary disease	9445	4.7	9448	4.7	0.982
Coronary artery disease	11068	5.5	11070	5.5	0.989
Diabetes mellitus	19987	9.9	19991	9.9	0.983
Hyperlipidemia	27114	13.4	27120	13.4	0.978
Hypertension	37919	18.8	37923	18.8	0.987

Data are presented as the number of individuals in each group, with percentages given in parentheses.

a Chi-square test.

b *t*-test comparing individuals with and without hyperthyroidism.

**Table 2:** Incidence density of herpes zoster between individuals with and without hyperthyroidism stratified by sex and age.

Hyperthyroidism					Non-hyperthyroidism						
	
Variable	N	Event	Person-years	Incidence^a^	N	Event	Person-years	Incidence^a^	IRR^b^	(95 % CI)	*P* value
All	202069	5007	821397	6.10	202069	8323	1508492	5.53	1.10	(1.07–1.14)	<0.001
Sex											
Male	49833	1100	196213	5.61	49833	1831	366561	5.00	1.12	(1.04–1.21)	0.003
Female	152236	3907	625183	6.25	152236	6492	1141931	5.69	1.10	(1.06–1.14)	<0.001
Age group (years)											
20–39	71808	736	302079	2.44	71792	1206	550981	2.19	1.11	(1.02–1.22)	0.022
40–64	105007	2960	428398	6.91	105014	5063	787616	6.44	1.07	(1.03–1.12)	0.002
65–84	25254	1311	90920	14.42	25263	2054	169892	12.11	1.19	(1.11–1.28)	<0.001

a Incidence: per 1000 person-years.

b IRR (incidence rate ratio): hyperthyroidism vs. non-hyperthyroidism (95 % confidence interval).

**Table 3:** Multivariable Cox model estimating hazard ratio and 95 % confidence interval of herpes zoster associated with hyperthyroidism and covariables.

Variable	Crude			Adjusted^a^		
	
	HR	(95 %CI)	*P* value	HR	(95 %CI)	*P* value
Sex (male vs. female)	0.89	0.85–0.92	<0.001	0.83	0.79–0.86	<0.001
Age group (years)						
20–39	Ref.			Ref.		
40–64	2.90	2.76–3.05	<0.001	2.72	2.59–2.86	<0.001
65–84	5.73	5.42–6.06	<0.001	4.56	4.27–4.86	<0.001
Hyperthyroidism (yes vs. no)	1.18	1.13–1.22	<0.001	1.19	1.15–1.23	<0.001
Baseline comorbidities (yes vs. no)						
Alcohol-related disease	0.83	0.58–1.18	0.303			
Cerebrovascular disease	1.82	1.68–1.97	<0.001	1.00	0.92–1.08	0.904
Chronic kidney disease	2.12	1.94–2.32	<0.001	1.18	1.08–1.30	<0.001
Chronic obstructive pulmonary disease	1.96	1.84–2.09	<0.001	1.38	1.30–1.48	<0.001
Coronary artery disease	2.12	2.00–2.24	<0.001	1.18	1.11–1.26	<0.001
Diabetes mellitus	1.87	1.79–1.96	<0.001	1.09	1.03–1.15	0.002
Hyperlipidemia	1.92	1.84–2.00	<0.001	1.16	1.11–1.22	<0.001
Hypertension	2.04	1.97–2.12	<0.001	1.13	1.08–1.19	<0.001

a Controlling for confounding variables including sex, age, cerebrovascular disease, chronic kidney disease, chronic obstructive pulmonary disease, coronary artery disease, diabetes mellitus, hyperlipidemia, and hypertension.
